# The impact of temperature and *Wolbachia* infection on vector competence of potential dengue vectors *Aedes aegypti* and *Aedes albopictus* in the transmission of dengue virus serotype 1 in southern Taiwan

**DOI:** 10.1186/s13071-017-2493-x

**Published:** 2017-11-07

**Authors:** Cheng-Hui Tsai, Tien-Huang Chen, Cheo Lin, Pei-Yun Shu, Chien-Ling Su, Hwa-Jen Teng

**Affiliations:** 1 Center for Diagnostics and Vaccine Development, Centers for Disease Control, Taipei, 11561 Taiwan, Republic of China; 2 Present address: Graduate Institute of Biomedical Sciences, Chang Gung University, Kwei-San, Tao-Yuan, 33332 Taiwan

**Keywords:** *Aedes aegypti*, *Aedes albopictus*, Temperature, *Wolbachia*, Dengue virus, Taiwan

## Abstract

**Background:**

We evaluated the impact of temperature and *Wolbachia* infection on vector competence of the local *Aedes aegypti* and *Ae. albopictus* populations of southern Taiwan in the laboratory.

**Results:**

After oral infection with dengue serotype 1 virus (DENV-1), female mosquitoes were incubated at temperatures of 10, 16, 22, 28 and 34 °C. Subsequently, salivary gland, head, and thorax-abdomen samples were analyzed for their virus titer at 0, 5, 10, 15, 20, 25 and 30 days post-infection (dpi) by real-time RT-PCR. The results showed that *Ae. aegypti* survived significantly longer and that dengue viral genome levels in the thorax-abdomen (10^3.25 ± 0.53^–10^4.09 ± 0.71^ PFU equivalents/ml) and salivary gland samples (10^2.67 ± 0.33^–10^3.89 ± 0.58^ PFU equivalents/ml) were significantly higher at high temperature (28–34 °C). The survival of *Ae. albopictus* was significantly better at 16 or 28 °C, but the virus titers from thorax-abdomen (10^0.70^–10^2.39 ± 1.31^ PFU equivalents/ml) and salivary gland samples (10^0.12 ± 0.05^–10^1.51 ± 0.31^ PFU equivalents/ml) were significantly higher at 22–28 °C. Within viable temperature ranges, the viruses were detectable after 10 dpi in salivary glands and head tissues in *Ae. aegypti* and after 5–10 dpi in *Ae. albopictus*. Vector competence was measured in *Ae*. *albopictus* with and without *Wolbachia* at 28 °C. *Wolbachia*-infected mosquitoes survived significantly better and carried lower virus titers than *Wolbachia*-free mosquitoes. *Wolbachia* coinfections (92.8–97.2%) with *w*AlbA and *w*AlbB strains were commonly found in a wild population of *Ae. albopictus*.

**Conclusions:**

In southern Taiwan, *Ae. aegypti* is the main vector of dengue and *Ae. albopictus* has a non-significant role in the transmission of dengue virus due to the high prevalence of *Wolbachia* infection in the local mosquito population of southern Taiwan.

**Electronic supplementary material:**

The online version of this article (10.1186/s13071-017-2493-x) contains supplementary material, which is available to authorized users.

## Background

Dengue fever is the most common arboviral disease in tropical and subtropical regions in the world. In recent decades, the numbers of dengue cases and countries with endemic dengue fever have dramatically increased because of global warming [1], rapid and frequent international traveling, ineffective vector control, changing life styles and unplanned urbanization [2]. The disease burden of dengue is estimated at 390 million dengue infections per year, with 96 million cases exhibiting apparent symptoms [3]. Moreover, a total of 3.9 billion people living in 128 countries have been identified as at risk for dengue virus infection [4]. In Taiwan, since 2004, outbreaks have occurred annually with peaks in 2014 and 2015 (15,492 and 43,419 indigenous cases, respectively). These local mosquito-transmitted cases were mainly found in southern Taiwan where *Aedes aegypti* L. and *Ae. albopictus* (Skuse) coexist. The former species has limited distributions in southern Taiwan, Taitung City in eastern Taiwan and the Penghu Islands. The latter species is found in islands below the altitudes of 1760 m above sea level [5].

Dengue outbreaks have occurred in late spring or early summer and have peaked in the fall in many countries, including Taiwan. Previous studies have found that temperature is the most important abiotic environmental factor because it affects the vector efficiency [6–8]. Viral replication and propagation speed are faster at higher temperatures. The extrinsic incubation period (EIP), the time from the initial acquisition of the dengue virus by mosquito vectors to the time of transmission to a human host ranges from 5 to 33 days at 25 °C and from 2 to 15 days at 30 °C, with an average of 15 and 6.5 days, respectively [9]. Additionally, the mosquito species and strain [10], virus serotypes and genotypes [11], virus uptake quantity [6], size of adult mosquitoes [12], and *Wolbachia* infection [13–15] also affect dengue viral replication or limit viral transmission. Among these factors, the effect of *Wolbachia* is worth paying attention to due to the disease control strategy. Although the mechanism of *Wolbachia*-mediated pathogen interference is unclear, *Wolbachia*-induced, density-dependent inhibition of dengue virus in *Aedes* mosquitoes is temperature sensitive [16]*.* Therefore, some studies showed no *Wolbachia* effect on chikungunya virus infection [17] and dengue virus [18] in *Ae. albopictus* mosquitoes, but other studies showed that *Wolbachia* infection reduced dengue viral transmission but not the viral load [14].

Dengue is not considered an endemic disease in Taiwan. Most dengue outbreaks have originated from imported dengue cases, in which the introduced virus was transmitted to local *Aedes* mosquitoes in the late spring or the early summer, except for four winter outbreaks in Taiwan. These outbreaks were DENV-1 in 1987–1988, DENV-2 in 2002–2003, DENV-3 in 2009–2010, and DENV-1 in 2014–2015 [19]. Furthermore, an ovitrap survey from 2010 to 2011 found that *Ae. aegypti* mosquitoes were able to oviposit all year long in Kaohsiung City [20]. The magnitude of dengue outbreaks can reach hundreds or thousands of people in the distribution areas of *Ae. aegypti*. However, only clusters (less than 20 dengue cases) of DENV-1 occurred in areas without the presence of *Ae. aegypti*, where *Ae. albopictus* mosquitoes served as the transmitting vectors instead. In Taiwan, *Wolbachia* infection with *w*AlbA and *w*AlbB is very common in the local *Ae. albopictus* population but not the *Ae. aegypti* population [21]. Therefore, the objective of this study was to compare the vector competence of local *Ae. aegypti* and *Ae. albopictus* populations in high-risk dengue areas in southern Taiwan for DENV-1 at different incubation temperatures in the laboratory. In addition, the natural effect of *Wolbachia* infection on the vector competence of the local *Ae. albopictus* population was also evaluated.

## Methods

### Mosquito collection and maintenance

*Aedes aegypti* and *Ae. albopictus* larvae and pupae were collected from a dengue high-risk area, the Chien-chen district, Kaohsiung City, in 2015 and 2016, respectively. Most collections occurred in the public domain and for any collections referred to the private sector, owners/residents were asked for permission to collect mosquitoes on their land/in their residences. The F1 adults used for the various temperature experiments, for each species, were from a single collection of larvae reared under the same conditions [20–30 °C with a photoperiod of 10:14 h (L:D)]. Immature mosquitoes were reared in a plastic pan (21 × 14 × 7 cm) containing 450 ml of deionized water. A sufficient amount of food (yeast extract and pig liver powder; 1:1 by weight) was provided daily. Adult mosquitoes were kept in an acrylic cage (29 × 20 × 20 cm) and were provided with a 10% sucrose solution. The first generation (F1) of these field populations was used in the following experiments. Additionally, to eliminate *Wolbachia* infection, adult F1 *Ae. albopictus* mosquitoes were fed a 1 mg/ml tetracycline solution in sucrose as described previously [22]. Each generation of mosquitoes was fed with tetracycline for at least two weeks and given a blood meal, and then, eggs hatched to produce the next generation (F1 to F3). When the third generation (F3) was shown to have no infection of *Wolbachia*, the fourth generation (F4) was used as the *Wolbachia*-free mosquitoes for further experiments. To minimize the impact of antibiotic on mosquito microflora, the eighth generation (F8) was also used as the *Wolbachia*-free mosquitoes for giving a recovery of 2 additional generations (tetracycline using in F1-F5).

### Experimental oral infection

Two- to five-day-old female mosquitoes were deprived of sugar solution for 24 h prior to the oral challenge. The feeding mixture was prepared by mixing equal parts of DENV-1-infected C6/36 supernatant and human blood treated with an anticoagulant (7 ml of human blood:18 mg of K2EDTA) (cat. no. 367525, Becton Dickinson and Company, New Jersey, USA). The virus strains (H1030440) used in this study was the dominant strain of 2014 [19], which was isolated from blood specimens of the indigenous cases. All were harvested at virus titer of 10^7^ plaque forming units (PFU) per ml (determined by plaque assay). A high virus titer of 1.63 × 10^7^ PFU/ml was used for the following experiments, and this titer was close to the high viral load (ranging from 1.9 × 10^6^ to 4.7 × 10^7^ PFU/ml) of viremic patients [23]. Ten females were placed in a small paper cup (8 cm diameter × 9.5 cm height) covered with fine nylon mesh. The Hemotek 5 W1 membrane feeding system (Hemotek Ltd., Lancashire, UK) was used and *Ae. aegypti* and *Ae. albopictus* mosquitoes were allowed to feed for 1 h. The fed mosquitoes were then held in a growth chamber at 10, 16, 22, 28 or 34 °C, with 75% relative humidity (RH). Cotton soaked in a 10% sugar solution was provided on the mesh and changed every 2 days. Mosquitoes were frozen at 0, 5, 10, 15, 20, 25, and 30 day intervals as knockouts. Then, the heads, salivary glands and thorax-abdomens were dissected for samples and stored at −80 °C until processing for virus or *Wolbachia* detection.

### Viral detection and titration

Individual samples (salivary glands, head, thorax-abdomen, midgut or fat-body) were homogenized and purified by centrifugation. Viral RNA (70 μl) was extracted from 140 μl of sample suspension using the QIAamp Viral RNA Mini Kit (cat. no. 52,906, Qiagen, Hilden, Germany) according to the manufacturer’s instructions. Amplification by RT-PCR was performed using the LightCycler quantitative PCR system (Roche Applied Science, California, USA). Samples were assayed in a 50 μl reaction mixture containing 10 μl of sample RNA and optimal concentrations of the primers using the QuantiTect SYBR Green RT-PCR Kit (cat. no. 204, 243, Qiagen, Hilden, Germany). Type I dengue virus-specific primers DN-F (5′-CAA TAT GCT GAA ACG CGA GAG AAA-3′) and D1-R (5′-CGC TCC ATA CAT CTT GAA TGA G-3′) [24], were used for real-time RT-PCR, and the products were expected to be 193 bp. The thermal profile consisted of a 30 min reverse transcription step at 50 °C, followed by 15 min of Taq polymerase activation at 95 °C; these steps were then followed by 45 cycles of PCR (94 °C for 15 s, annealing temperature 55 °C for 30 s, and 72 °C for 20 s).

Additionally, serial ten-fold dilutions of dengue virus (strain H1030440) were performed with an initial viral load of 1.63 × 10^7^ PFU/ml. Each dilution was added to 140 μl of the salivary glands, heads, thorax-abdomens, midguts and fat-body sample suspension of one non-infected *Ae. aegypti* or *Ae. albopictus* female mosquito to estimate the virus titers in the salivary glands, heads, thorax-abdomens, midguts and fat-body of orally infected mosquitoes. The virus titer standard curve was generated by real-time RT-PCR. The linear regressions of the Ct value (Y) against log (viral load, Z) in the salivary glands, heads, thorax-abdomens, midgut and fat-body of *Ae. aegypti* females were Y = -4.394 × log(Z) + 49.50 (R^2^ = 0.9985; salivary glands); Y = -3.985 × log(Z) + 46.61 (R^2^ = 0.9984; heads); Y = -3.823 × log(Z) + 46.49 (R^2^ = 0.9985; thorax-abdomens); Y = -4.536 × log(Z) + 46.29 (R^2^ = 0.9995; midguts); and Y = -4.029 × log(Z) + 45.68 (R^2^ = 0.9999; fat-body), respectively. linear regressions of the Ct value (Y) against log (viral load, Z) in the salivary glands, heads, thorax-abdomens, midgut and fat-body of *Ae. albopictus* females were Y = -3.321 × log(Z) + 38.66 (R^2^ = 0.9968; salivary glands); Y = -3.988 × log(Z) + 41.77 (R^2^ = 0.9943; heads); Y = -3.993 × log(Z) + 44.50 (R^2^ = 0.9975; thorax-abdomens); Y = -4.512 × log(Z) + 47.27 (R^2^ = 0.9991; midguts); and Y = -4.130 × log(Z) + 44.12 (R^2^ = 0.9995; fat-body), respectively. For each sample, the viral titer was calculated according to the linear regression above.

### *Wolbachia* detection

The first generation (F1) of *Ae. albopictus* adults was used to detect *Wolbachia* infection. DNA was extracted from the mosquitoes used in the previous experiments by the QIAamp® DNA Mini Kit (cat. no. 51306, Qiagen, Hilden, Germany). The PCR reaction was conducted as described by previous studies [25]. Two primer pairs (328F, 5′-CCA GCA GAT ACT ATT GCG-3′, and 691R, 5′-AAA AAT TAA ACG CTA CTC CA-3′; 183F, 5′-AAG GAA CCG AAG TTC ATG-3′ and 691R) were used to amplify and detect *Wolbachia* surface protein genes (wsp), group A *w*AlbA (501 bp) and group B *w*AlbB (379 bp) in this study. Universal primers (12SRNA*-*Forward, 5′-AAA CTA GCA TTA GAT ACC CTA TTA T-3′ and 12SRNA*-*Reverse, 5′-AAG AGC GAC GGG CGA TGT GT-3′) were used to amplify a cDNA fragment of the insect mtDNA (12SRNA gene), as a control to assess the quality of the template DNA extracted from mosquitoes.

### Quantification of *Wolbachia* density

The density of *Wolbachia* in the first generation (F1) of *Ae. albopictus* adults reared at 28 °C and different temperatures was determined for the *w*AlbA and *w*AlbB infections. Real-time PCR was used for the quantification of the *Wolbachia* gene (*w*AlbA and *w*AlbB) as described by [18, 26]. The *w*AblA and *w*AlbB gene was normalized to the mosquito housekeeping gene mRpS6 to adjust for different tissues. Primer pairs (*w*AlbA, F-5′-GGG TTG ATG TTG AAG GAG-3′ and R-5′-CAC CAG CTT TTA CTT GAC C-3′; *w*AlbB, F-5′-CCT TAC CTC CTG CAC AAC AA-3′ and R-5′-GGA TTG TCC AGT GGC CTT A-3′; mRpS6, F-5′-AGT TGA ACG TAT CGT TTC CCG CTA C-3′ and R-5′-GAA GTG ACG CAG CTT GTG GTC GTC C-3′) were used. The qPCR cycling conditions used are as follows: an initial incubation at 90 °C for 5 min, followed by 45 cycles of amplification at 95 °C for 10 s, 56 °C for 20 s and 72 °C for 20 s and a melting curve detection and a final cooling step of 40 °C for 10 s. The relative quantification fold of *Wolbachia* was calculated using the following formula 2^ -(ct of *w*Alb/ct of mRpS6).

### Statistical analysis

Statistical analyses were performed with Statistica 10 software (StatSoft, Inc., Tulsa, OK, USA). Initially, we performed a test to compare the five survival curves against dpi for each mosquito species using the non-parametric Kaplan-Meier analysis [27]. If this test revealed significant differences among all curves for each species, we then ran a paired log-rank test to identify which paired curves were different. Mosquitoes that were killed to detect the virus for each dpi category were classified as censored observations [28]. Additionally, a paired log-rank test was used to compare survival against dpi for *Wolbachia*-infected and *Wolbachia*-free mosquitoes of *Ae. albopictus*. Because of the small number of samples tested for each combination, Fisher’s exact test was used to test the differences in thorax-abdomen and salivary gland infection rates. The Kolmogorov-Smirnov test was used to compare the overall differences in the entire growth curve of the virus at different temperatures in the thorax-abdomen (data on viral uptake at 0 dpi were excluded), salivary glands, and head samples. Student’s t tests were used to detect the differences in virus titers between mosquito species, incubation temperatures and dpi.

## Results

### Virus detection in *Ae. aegypti*

**Fig. 1:**
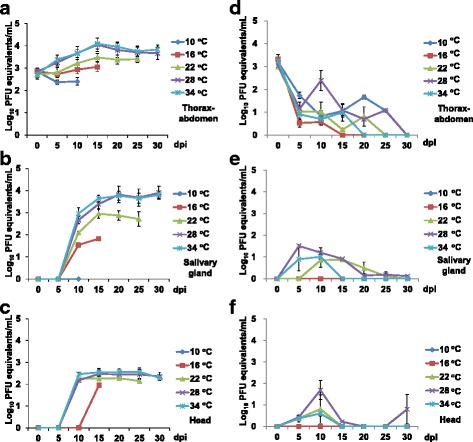
Virus titers (mean ± SE) in the thorax-abdomen (**a**), salivary gland (**b**) and head (**c**) samples of infected *Aedes aegypti* mosquitoes and the thorax-abdomen (**d**), salivary gland (**e**) and head (**f**) samples of infected *Aedes albopictus* mosquitoes at different incubation temperatures with an initial virus titer of 1.63 × 10^7^ PFU/ml (initial *n* = 10 for each combination of temperature, dpi, and mosquito species)

Infection rates (range 60–100%) of F1 field collected *Ae. aegypti* mosquitoes after oral infection with DENV-1 were unrelated to incubation temperatures (Fisher’s exact test, *P* > 0.05) (Table 1). From the 0 dpi data, all female mosquitoes were 100% infected with no significant difference (*P* > 0.05) in virus titers (10^2.70 ± 0.56^–10^2.86 ± 0.40^ PFU equivalents/ml) (Fig. 1a). Viral loads were dependent on incubation temperatures (Fig. 1a). The virus growth curves of infected *Ae. aegypti* at higher temperatures (28 and 34 °C) were not significantly different (Kolmogorov-Smirnov test, *P* > 0.05) but were significantly different (Kolmogorov-Smirnov test, *P* < 0.05) from other temperatures (10, 16 and 22 °C). When incubated at 34 °C, the level of viral genomes was significantly (t-test, *t* = 2.38–4.12, *df* = 8–11, *P* < 0.05) higher than at 10 °C and 5–10 dpi, at 16 °C and 5–15 dpi or at 22 °C and 20 dpi. At 22–34 °C, the viral loads showed an increasing trend with a peak at 15 dpi. The peak viral titers at 22, 28 and 34 °C were 10^3.49 ± 0.47^, 10^4.05 ± 0.86^ and 10^4.09 ± 0.71^ PFU equivalents/ml, respectively (Additional file 1: Table S1).

**Table 1:** Mosquito infection rates (%) with days post-infection (dpi) at different incubation temperatures (number of thorax-abdomen samples testing positive for virus genomes/total number of samples)

Species	dpi	10 °C	16 °C	22 °C	28 °C	34 °C
*Ae. aegypti*	0	100 (10/10)	100 (10/10)	100 (10/10)	100 (10/10)	100 (10/10)
	5	100 (6/6)	86 (6/7)	88 (7/8)	88 (7/8)	88 (7/8)
	10	100 (4/4)	100 (6/6)	88 (7/8)	100 (8/8)	88 (7/8)
	15		100 (5/5)	100 (6/6)	88 (7/8)	88 (7/8)
	20			100 (4/4)	100 (8/8)	86 (6/7)
	25			100 (2/2)	100 (7/7)	86 (6/7)
	30				100 (6/6)	60 (3/5)
*Ae. albopictus*	0	100 (10/10)	100 (10/10)	100 (10/10)	100 (10/10)	100 (10/10)
	5	100 (9/9)	89 (8/9)	100 (9/9)	90 (9/10)	78 (7/9)
	10	88 (7/8)	44 (4/9)	75 (6/8)	90 (9/10)*	38 (3/8)*
	15	57 (4/7)*	0 (0/9)*	13 (1/8)*	67 (6/9)*	60 (3/5)
	20	40 (2/5)	0 (0/9)	33 (2/6)	13 (1/8)	0 (0/3)
	25	25 (1/4)	0 (0/8)	20 (1/5)	14 (1/7)	0 (0/3)
	30	0 (0/3)	0 (0/7)	0 (0/4)	0 (0/5)	0 (0/1)

^*^Infection rates of *Aedes albopictus* at 10 dpi with incubation at 28 °C were significantly higher than those at 34 °C, as determined by Fisher’s exact test. Significant differences were also observed at 15 dpi between 10 °C and 16 °C and between 28 °C and 16 or 22 °C

The rate or titer of viral infection of the salivary gland is known as an indicator of transmission potential. After 10 dpi, higher salivary gland infection rates (range 25–100%) at 22–34 °C were found compared with the lower incubation temperatures (range 0–20%), although no significant differences were detected (Fisher’s exact, *P* > 0.05) (Table 1). In the salivary gland samples of *Ae. aegypti*, no viral genomes were detected at 10 °C or at 0–5 dpi and 16–34 °C (Fig. 1b, Table 2). The viral load increased with infection time after 10 dpi. The virus growth curves of infected *Ae. aegypti* at higher temperatures (28 and 34 °C) were not significantly different from each other (Kolmogorov-Smirnov test, P > 0.05) but were significantly different (Kolmogorov-Smirnov test, *P* < 0.05) from that at low temperatures (10, and 16 °C). The growth curve at 22 °C was not significantly different (Kolmogorov-Smirnov test, *P* > 0.05) from that at 28 °C but was significantly different (Kolmogorov-Smirnov test, *P* < 0.05) from that of 34 °C. Significant differences (t-test, *t* = 2.94–4.44, *df* = 5, *P* < 0.05) in virus titer were detected at 15 dpi between 16 °C and higher (28 or 34 °C) incubation temperatures. Additionally, the viral loads at 15–20 dpi and 34 °C or at 15 dpi and 28 °C were significantly higher (t-test, *t* = 2.43–3.85, *df* = 6–9, *P* < 0.05) than those at 22 °C. At 16 °C, only viral genomes were detected in one female at 10 and 15 dpi with viral loads of 10^1.54^ and 10^1.82^ PFU equivalents/ml, respectively. At 22 °C, viral genomes were detected in the salivary glands of 2 females at 10 dpi with 10^2.09 ± 0.08^ PFU equivalents/ml of the viral load. The viral loads increased to 10^2.69 ± 0.43^, 10^2.54 ± 0.31^, and 10^2.38^ PFU equivalents/ml at 15, 20 and 25 dpi, respectively, with a salivary gland infection rate range of 83–100%. At 28 and 34 °C, viral genomes were first detected in the salivary gland samples at 10 dpi and viral loads of 10^2.67 ± 0.33^ and 10^2.96 ± 0.52^ PFU equivalents/ml (salivary gland infection rates = 50–63%). At 15–30 dpi, the viral loads detected were between 10^3.38 ± 0.49^ and 10^3.89 ± 0.58^ PFU equivalents/ml with a salivary gland infection range of 57–88%. In the head samples, viral genomes were first detected at 16 °C and 15 dpi with a quantity of 10^1.95^ PFU equivalents/ml (Fig. 1c). At 22–34 °C, virus genomes were first detected at 10 dpi and were maintained as constant titers thereafter. Viral titers of viruses grown at low (10 or 16 °C) and high (28 or 34 °C) incubation temperatures at 10 dpi were significantly different (t-test, *t* = 3.05–55.33, *df* = 5–8, *P* < 0.05).

**Table 2:** Mosquito salivary gland infection rates (%) with days post-infection (dpi) at different temperatures (number of salivary gland samples testing positive for virus genomes/total number of samples)

Species	dpi	10 °C	16 °C	22 °C	28 °C	34 °C
*Ae. aegypti*	0	0 (0/10)	0 (0/10)	0 (0/10)	0 (0/10)	0 (0/10)
	5	0 (0/6)	0 (0/7)	0 (0/8)	0 (0/8)	0 (0/8)
	10	0 (0/4)	17 (1/6)	25 (2/8)	50 (4/8)	63 (5/8)
	15		20 (1/5)	83 (5/6)	88 (6/8)	88 (6/8)
	20			100 (4/4)	63 (5/8)	57 (4/7)
	25			100 (2/2)	57 (4/7)	57 (4/7)
	30				67 (4/6)	60 (3/5)
*Ae. albopictus*	0	0 (0/10)	0 (0/10)	0 (0/10)	0 (0/10)	0 (0/10)
	5	0 (0/9)	0 (0/9)	0 (0/9)	40 (4/10)	22 (2/9)
	10	0 (0/8)^a^	0 (0/9)^a^	75 (6/8)^a^	70 (7/10)^a^	38 (3/8)
	15	0 (0/7)	0 (0/9)	13 (1/8)	11 (1/9)	0 (0/5)
	20	0 (0/5)	0 (0/9)	33 (2/6)	13 (1/8)	0 (0/3)
	25	0 (0/4)	0 (0/8)	20 (1/5)	29 (2/7)	0 (0/3)
	30	0 (0/3)	0 (0/7)	0 (0/4)	40 (2/5)	0 (0/1)

^a^Infection rates at 10 dpi with incubation at 22 or 28 °C were significantly (*P* < 0.01 or 0.05) higher than those at 10 or 16 °C, as determined by Fisher’s exact test

### Virus detection in *Ae. albopictus*

Infection rates in *Ae. albopictus* at 10 dpi with incubation at 28 °C were significantly (Fisher’s exact test, *P* < 0.05) higher than at 34 °C. Significant differences (Fisher’s exact test, *P* < 0.05) were also found at 15 dpi between 10 °C and 16 °C and between 28 °C and 16 or 22 °C (Fig. 1d). No viruses were detected in the salivary gland (Fig. 1e) or head samples (Fig. 1f) at 10–16 °C or at 0–5 dpi and 22–34 °C. The highest salivary gland infection rates (38–75%) were detected at 10 dpi for 22–34 °C incubation temperatures and the rates at 22 or 28 °C were significantly (Fisher’s exact test, *P* < 0.05) higher than those at lower temperatures (10 or 16 °C). From the 0 dpi data, all female mosquitoes were 100% infected without significant difference (t-test, *t* = 0–1.56, *df* = 18, *P* > 0.05) in virus titers (10^2.98 ± 0.30^–10^3.33 ± 0.64^ PFU equivalents/ml) (Fig. 1d). Viral genomes were detected in the thorax-abdomen samples with low titers (10^0.23^–10^2.39 ± 1.31^ PFU equivalents/ml) at all incubation temperatures (10–34 °C) after 10 dpi with largely varied infection rates (0–90%) (Table 1). Viral genomes were first detected at 10 dpi and 22 °C and earlier (5 dpi) at higher temperatures (28 or 34 °C) with low titers (10^0.12 ± 0.05^–10^1.51 ± 0.31^ PFU equivalents/ml). In the head samples, viral genomes were detected at 5–30 dpi with low titers (10^0.22^–10^1.69 ± 1.32^ PFU equivalents/ml) in 22–34 °C environments.

### Impact of *Wolbachia* on *Ae. albopictus*

**Fig. 2:**
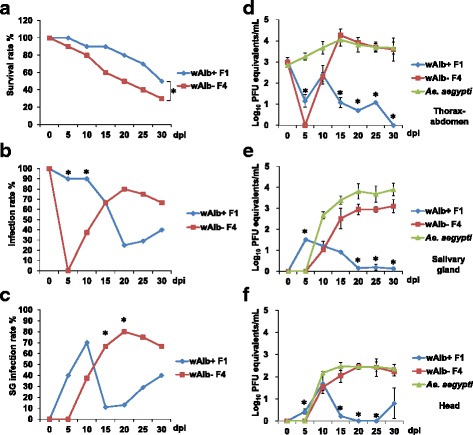
Comparison of *Wolbachia*-infected (F1) and *Wolbachia*-free (F4) *Aedes albopictus* mosquitoes at 28 °C by survival rate (**a**), thorax-abdomen infection rate (**b**) and salivary gland infection rate (**c**). Virus titers (mean ± SE) detected in thorax-abdomen (**d**), salivary glands (**e**) and head (**f**) samples with an initial virus titer of 1.63 × 10^7^ PFU/ml (removal of *Wolbachia* by tetracycline for 3 generations; *n* = 10 for each dpi; * indicates significant difference by a Fisher’s exact test or a t-test at *P* < 0.05)

Since lower viral replication was found in *Ae. albopictus* compared with *Ae. aegypti* mosquitoes, we analyzed the infection rate of *Wolbachia* in the tested *Ae. albopictus* mosquitoes. Interestingly, the results showed that 92.8–97.2% of *Ae. albopictus* mosquitoes were co-infected with *Wolbachia* group A (*w*AlbA) and group B (*w*AlbB) (Table 3). Only 1.4–2.9% of females were not infected with *Wolbachia*. To determine whether *Wolbachia* in *Ae. albopictus* affects DENV-1 replication, we generated *Wolbachia-*free mosquitoes with tetracycline treatment in 3 generations (Additional file 1: Figure S1). We fed *Wolbachia-*free mosquitoes with a blood-meal with the same DENV-1 and then incubated them at 28 °C, which has the best viral load among the tested temperatures. The survival trend of *Wolbachia-*free *Ae. albopictus* mosquitoes fed with DENV-1 significantly (log-rank test, *P* < 0.05) decreased compared with *Wolbachia*-infected mosquitoes (Fig. 2a). The infection rate in *Wolbachia-*free *Ae. albopictus* mosquitoes increased from 5 to 20 dpi and then slightly dropped (Fig. 2b). The infection rate in the *Wolbachia*-infected mosquitoes was significantly higher at 5 (Fisher’s exact test, *P* < 0.01) and 10 dpi (Fisher’s exact test, *P* < 0.05) than in the *Wolbachia-*free mosquitoes. We also found the same transmission trend (increasing from 5 to 20 dpi and then dropping slightly) in the *Wolbachia*-free mosquitoes. The salivary gland infection rate of the *Wolbachia-*free mosquitoes was significantly (Fisher’s exact test, *P* < 0.05) higher at 15 and 20 dpi compared to *Wolbachia-*infected mosquitoes at 28 °C (Fig. 2c). We also found that the salivary glands of *Wolbachia*-infected mosquitoes became infected earlier at 5 dpi than those of the *Wolbachia*-free mosquitoes at the margin of significance (Fisher’s exact test, *P* = 0.0542).

**Table 3:** *Wolbachia* infection in the tested *Aedes albopictus* mosquitoes at different temperatures

Incubation temperatures	No. of mosquitoes tested	Non-infected		*w*AlbA only		*w*AlbB only		Coinfection with A and B	
		*n*	%	*n*	%	*n*	%	*n*	%
10 °C	70	1	1.4	1	1.4	0	0	68	97.2
16 °C	70	2	2.9	2	2.9	1	1.4	65	92.8
22 °C	70	2	2.9	1	1.4	1	1.4	66	94.3
28 °C	70	1	1.4	1	1.4	0	0	68	97.2
34 °C	70	2	2.9	3	4.3	0	0	65	92.8

The virus growth curves in thorax-abdomen and salivary gland samples of *Wolbachia*-free *Ae. albopictus* and *Ae. aegypti* were significantly different from those of *Wolbachia*-infected *Ae. albopictus* (Kolmogorov-Smirnov test, *P* < 0.05) but not significantly different (Kolmogorov-Smirnov test, *P* > 0.05) from each other. Viral genomes were first detected in the thorax-abdomen, salivary gland, and head samples of the *Wolbachia*-free mosquitoes at 10 dpi with viral titers 10^2.29 ± 0.37^, 10^1.02 ± 0.12^ and 10^1.52 ± 0.26^ PFU equivalents/ml, respectively (Fig. 2d-f). The virus titer in the thorax-abdomen samples peaked (10^4.26 ± 0.58^ PFU equivalents/ml) at 15 dpi and remained high (Fig. 2d). Virus titers of *Wolbachia*-free mosquitoes were significantly higher at 15–30 dpi (t test, *P* < 0.05) and lower at 5 dpi (t-test, *t* = 3.46, *df* = 17, *P* < 0.01) compared with the *Wolbachia*-infected mosquitoes. Viral genomes were detected in the salivary gland samples at 10–30 dpi with increasing titers (10^1.02 ± 0.12^–10^3.11 ± 0.45^ PFU equivalents/ml) (Fig. 2e). Virus titers of *Wolbachia*-free mosquitoes were significantly higher at 20–30 dpi (t-test, *t* = 5.00–14.01, df = 2–3, *P* < 0.05) and lower at 5 dpi (t-test, *t* = 48.10, *df* = 11, *P* < 0.01) compared with the *Wolbachia*-infected mosquitoes. Virus titers in head samples were detected from 10 to 30 dpi at quantities of 10^1.52 ± 0.26^–10^2.22 ± 0.26^ PFU equivalents/ml (Fig. 2f and Additional file 1: Table S2). The virus titers of *Wolbachia*-free mosquitoes were significantly higher (t test, *P* < 0.01) at 15–25 dpi and lower (t-test, *t* = 2.51, *df* = 17, *P* < 0.05) at 5 dpi compared with those of the *Wolbachia*-infected mosquitoes.

**Fig. 3:**
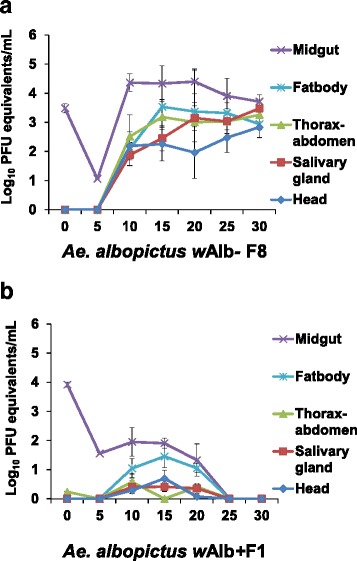
Virus titers (mean ± SE) in the thorax-abdomen, salivary gland, head, midgut and fat-body samples of *Wolbachia*-free (F8) (**a**), and *Wolbachia*-infected (F1) (**b**) of *Aedes albopictus* with an initial virus titer of 1.63 × 10^7^ PFU per ml (removal of *Wolbachia* by tetracycline for 5 generations and recovery for 2 generations; *n* = 10 for each dpi)

To minimize the impact of the antibiotic on mosquito microflora, the eighth generation (F8, tetracycline was stopped at F6) was also evaluated as the *Wolbachia*-free mosquitoes at 28 °C condition. Furthermore, insect midgut and fat-body is highly related to the immune defense system for pathogens. We also detected the dengue viral genome copy in these two samples. High viral genome copies were detected in midgut samples, followed by fat-body, salivary gland, thorax-abdomen, and head samples in *Wolbachia*-free (F8) from 10 to 30 dpi (Fig. 3a). Only few viruses could be detected in all samples of *Wolbachia*-infected (F1) *Ae. albopictus* from 10 to 20 dpi, and no virus could be detected after 25 dpi (Fig. 3b).

**Fig. 4:**
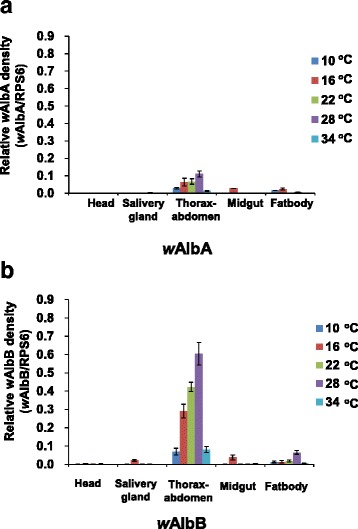
*Wolbachia* relative densities (ratio of *w*AlbA or *w*AlbB to host rps6 genomes using the formula 2^ -(ct of wAlb/ct of mRpS6) of *w*AlbA (**a**) and *w*AlbB (**b**) at different tissues (including head, thorax-abdomen, salivary gland, midgut and fat-body) and temperatures (10, 16, 22, 28 and 34 °C)*.* Bars around the mean values represent standard error (SE)

*Wolbachia* can induce density-dependent inhibition of dengue virus in *Aedes* mosquitoes, and its density is temperature sensitive. *Wolbachia* density was also measured in different tissues at different temperatures in *Wolbachia*-infected F1 *Ae. albopictus* (Fig. 4). *w*AlbB (Fig. 4a) had higher densities than *w*AlbA (Fig. 4a). The relative density of *w*AlbA and *w*AlbB (ratio of *w*AlbB or *w*AlbA to host rps6 genomes) was commonly found in thorax-abdomen samples (0.1104 ± 0.0179 and 0.6048 ± 0.0617, respectively), followed by fat-body (0.0052 ± 0.0017 and 0.0652 ± 0.0103, respectively) and midgut samples (0.0003 ± 0.0002 and 0.0008 ± 0.0001, respectively). For thorax-abdomen samples, the temperature-dependent effect from 10 to 28 °C on *Wolbachia* density was found in both strains. The results showed that both *w*AlbA (Fig. 4a) and *w*AlbB (Fig. 4b) were significantly located in thorax-abdomen compared to other tissues and had the highest density at the 28 °C rearing condition. At 16 °C and 22 °C, *Wolbachia* density was also detected more than at 10 °C and 34 °C. Additionally, the density of wAlbB was higher than that of wAlbA in all tissues of *Ae. albopictus* (2.4–6.3 times in thorax-abdomen samples and over 12.4 times in the fat-body).

### Survival rates of DENV-1-infected *Ae. aegypti* and *Ae. albopictus* mosquitoes

**Fig. 5:**
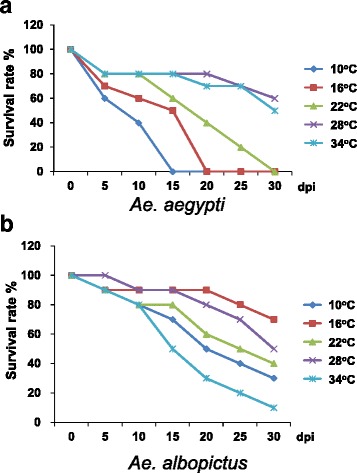
Mosquito survival rates of *Aedes aegypti* (**a**) and *Aedes albopictus* (**b**) with days post-infection (dpi) at different incubation temperatures (*n* = 10 for each combination of temperature, dpi, and mosquito species)

The mosquito survival trends of infected *Ae. aegypti* were significantly different (*χ* ^2^ = 28.8, *df* = 4, *P* = 0.00001) among the 5 incubation temperatures (Fig. 5a). Mosquitoes survived significantly better (log-rank test, *P* < 0.01) at 28 and 34 °C. No difference (log-rank test, *P* > 0.05) was found between these two incubation temperatures. The mosquito survival rate of infected *Ae. aegypti* at 10 °C was 40% at 10 dpi, which fell to 0 at 15 dpi (Fig. 5a). At 16 °C, the survival rates slightly increased; the survival rates were 50% and 0% at 15 dpi and 20 dpi, respectively. At 22 °C, the survival rates were 60%, 20% and 0% at 15, 25 and 30 dpi, respectively. At 28 °C and 34 °C, the survival rates were 80% and 50–60% at 25 and 30 dpi, respectively.

The mosquito survival trend of infected *Ae. albopictus* mosquitoes was significantly different (*χ* ^2^ = 15.8, *df* = 4, *P* = 0.00337) among the 5 incubation temperatures (Fig. 5b). Mosquitoes survived significantly better (log-rank test, test statistic = 2.28–4.21, *P* < 0.05) at 16 or 28 °C than at other incubation temperatures, but no difference (log-rank test, test statistic = 0.73, *P* > 0.05) was found between these 2 temperatures. Additionally, no differences (log-rank test, test statistic = 1.64, *P* > 0.05) were found in the trend of survival curves between 22 and 28 °C. At 16 °C, the survival rates decreased to 90%, 80%, and 70% at 10, 20 and 30 dpi, respectively. At 22 and 28 °C, the survival rates were 80–88%, 60–80% and 40–50% in the same 3 periods. At 34 °C, the mosquitoes showed the poorest survival, with survival rates of 10% at 30 dpi. In addition, the *Wolbachia*-free *Ae. albopictus* mosquitoes survived significantly better (log-rank test, test statistic = −2.51, *P* < 0.05) than the *Wolbachia*-infected mosquitoes (Fig. 2a).

## Discussion

Our data confirmed the temperature effects on virus replication as in previous studies, but we detected the native *Wolbachia* effect on *Ae. albopictus* vector competence by increasing DENV replication in *Wolbachia*-cured *Ae. albopictus*. *Aedes aegypti* mosquitoes survived better, and virus titers were significantly higher at high temperature (28 or 34 °C). However, *Ae. albopictus* mosquitoes lived longer at 16 and 28 °C, and virus titers were significantly higher at 22 and 28 °C (Fig. 1a, b, d, e and Fig. 5). Viruses were first detected at 10 dpi in salivary glands and head tissues in *Ae. aegypti* and 5 or 10 dpi in *Ae. albopictus* (Fig. 1b-d, f). *Wolbachia* infections were detected in up to 97% of the tested *Ae. albopictus* mosquitoes (Table 3). We concluded that in southern Taiwan, *Ae. aegypti* is the main vector of dengue and *Ae. albopictus* has an insignificant role due to the high native *Wolbachia* infection in the local mosquito population. Therefore, the elimination or a significant reduction of *Ae. aegypti* populations is an effective method for dengue prevention in southern Taiwan, where *Ae. aegypti* and *Ae. albopictus* coexist.

The EIP is known to be temperature dependent [6, 25]. Within viable temperature ranges, a higher temperature is associated with a shorter EIP. In our study, when incubation temperatures were greater than 22 °C, the EIP in *Ae aegypti* for the dengue virus was between 5 and 10 days, regardless of the incubation temperature. These results were confined to our mosquito collection setting at 5 dpi intervals with a fixed virus uptake quantity, which was not sensitive for the detection of the EIP temperature-dependent trend in *Ae. aegypti*. However, this trend was clearly detected in the *Ae. albopictus* species, in which EIPs (between 0 and 5 days) were shorter at higher temperatures (28 and 34 °C) and longer (between 5 and 10 days) at 22 °C (Fig. 1e). The thresholds for dengue transmission in constant temperatures were 13 °C [29] and 35 °C [6, 30], which was consistent with our results in *Ae. aegypti* (Fig. 1b, c), no virus particles in salivary glands and head samples were found at 10 °C). Recent studies have simulated diurnal field temperature fluctuations to show that this factor could shorten the extrinsic incubation period [31–33].

In our study, the survival rates of *Ae. albopictus* mosquitoes infected with *Wolbachia* were significantly better (Fig. 5). Although the study of Mousson et al. showed the same survival trend, no significant difference was detected [14]. This outcome might be due the different virus strain infected (DENV-1 *vs* DENV-2) or mosquito strain. This study found that local *Ae. albopictus* has a high percentage of infection with *w*AlbA and *w*AlbB (Table 3). In Taiwan, 51.7% of mosquitoes in 29 species were infected with *Wolbachia*, and co-infections with *w*AlbA and *w*AlbB have also been recorded [21]. However, none of the *Ae. aegypti* mosquitoes (the first generation of field collected larvae, *n* = 32) were infected with *Wolbachia* in this study.

*Wolbachia*-free (F4 and F8) *Ae. albopictus* mosquitoes showed sufficient vector competence after orally fed dengue virus compared to original mosquitoes (F1) and *Ae. aegypti*, indicating that *Wolbachia* infection may play a role in *Ae. albopictus* transmission of the dengue virus. In our study, we showed the reduction of virus load in all tissue samples (Figs. 2, 3), which was inconsistent with other natural *Wolbachia* infection studies. In these studies, either no native *Wolbachia* effect [18] or only virus transmission reduction [14] was found in dengue virus in *Ae. albopictus* mosquitoes.

Altogether, we showed that low temperatures limit the vector competence of local *Ae. aegypti* and *Ae. albopictus* populations in the transmission of DENV-1. Furthermore, the highly native *Wolbachia* infection was able to reduce viral titers and limit the transmission of DENV-1 in the local *Ae. albopictus* population.

## Conclusions

In southern Taiwan, *Ae. aegypti* is the main vector of dengue and *Ae. albopictus* has a non-significant role in the transmission of dengue virus due to the high prevalence of *Wolbachia* infection in the local mosquito population of southern Taiwan.

## Additional file

Additional file 1:
**Table S1.** Details of the virus titers of tested F1 mosquitoes. **Table S2.** Details of the virus titers of tested *Wolbachia* free F4 mosquitoes. **Figure S1.** Generation of the *Wolbachia*-free *Ae. albopictus* mosquitoes (DOCX 112 kb)
