# Supplementary material for: The impact of temperature and Wolbachia infection on vector competence of potential dengue vectors Aedes aegypti and Aedes albopictus in the transmission of dengue virus serotype 1 in southern Taiwan
Source: Parasit Vectors. 2017 Nov 7;10:551. doi: 10.1186/s13071-017-2493-x (PMC5678766; doi:10.1186/s13071-017-2493-x)
Supplement: Additional file 1: — Table S1. Details of the virus titers of tested F1 mosquitoes. Table S2. Details of the virus titers of tested Wolbachia free F4 mosquitoes. Figure S1. Generation of the Wolbachia-free Ae. albopictus mosquitoes (DOCX 112 kb) [file 13071_2017_2493_MOESM1_ESM.docx]

**Additional file 1: Table S1** Detail virus titers of tested F1 mosquitoes

| Samples tested | Virus titers (mean±SD) | | | | | | | | | | |
| --- | --- | --- | --- | --- | --- | --- | --- | --- | --- | --- | --- |
|  | dpi | 10°C | | 16°C | | 22°C | | 28°C | | 34°C | |
| Thorax-abdomen of F1 field-collected *Aedes aegypti* | 0 | 10^2.84±0.41^ | | 10^2.86±0.40^ | | 10^2.70±0.56^ | | 10^2.84±0.33^ | | 10^2.72±0.44^ | |
|  | 5 | 10^2.36±0.26^ | | 10^2.73±0.21^ | | 10^2.80±0.53^ | | 10^3.25±0.53^ | | 10^3.38±0.58^ | |
|  | 10 | 10^2.41±0.33^ | | 10^2.81±0.41^ | | 10^3.21±0.70^ | | 10^3.67±0.73^ | | 10^3.66±0.54^ | |
|  | 15 | - | | 10^2.93±0.43^ | | 10^3.49±0.47^ | | 10^4.05±0.86^ | | 10^4.09±0.71^ | |
|  | 20 | - | | - | | 10^3.01±0.15^ | | 10^3.81±0.66^ | | 10^3.96±0.40^ | |
|  | 25 | - | | - | | 10^3.25^ | | 10^3.70±0.63^ | | 10^3.75±0.58^ | |
|  | 30 | - | | - | | - | | 10^3.66±0.63^ | | 10^3.83±0.35^ | |
| Salivary glands of F1 field-collected *Aedes aegypti* | 0 | - | | - | | - | | - | | - | |
|  | 5 | - | | - | | - | | - | | - | |
|  | 10 | - | | 10^1.54^ | | 10^2.09±0.08^ | | 10^2.67±0.33^ | | 10^2.96±0.52^ | |
|  | 15 | - | | 10^1.82^ | | 10^2.69±0.43^ | | 10^3.38±0.49^ | | 10^3.64±0.37^ | |
|  | 20 | - | | - | | 10^2.54±0.31^ | | 10^3.81±0.82^ | | 10^3.76±0.41^ | |
|  | 25 | - | | - | | 10^2.38^ | | 10^3.68±0.77^ | | 10^3.65±0.44^ | |
|  | 30 | - | | - | | - | | 10^3.89±0.58^ | | 10^3.80±0.26^ | |
| Head of F1 field-collected *Aedes aegypti* | 0 | - | | - | | - | | - | | - | |
|  | 5 | - | | - | | - | | - | | - | |
|  | 10 | - | | - | |  | | 10^2.17±0.13^ | | 10^2.46±0.11^ | |
|  | 15 | - | | 10^1.95^ | |  | | 10^2.47±0.44^ | | 10^2.54±0.35^ | |
|  | 20 | - | | - | |  | | 10^2.44±0.31^ | | 10^2.56±0.31^ | |
|  | 25 | - | | - | |  | | 10^2.44±0.45^ | | 10^2.56±0.37^ | |
|  | 30 | - | | - | |  | | 10^2.35±0.43^ | | 10^2.39±0.03^ | |
| Thorax-abdomen of F1 field-collected *Aedes albopictus* | 0 | | 10^3.19±0.42^ | | 10^3.33±0.64^ | | 10^2.98±0.30^ | | 10^3.04±0.51^ | | 10^3.24±0.42^ |
|  | 5 | | 10^1.73±0.41^ | | 10^0.52±0.48^ | | 10^1.02±0.10^ | | 10^1.15±0.87^ | | 10^0.90±0.63^ |
|  | 10 | | 10^0.82±0.62^ | | 10^0.57±0.16^ | | 10^1.01±1.02^ | | 10^2.39±1.31^ | | 10^0.70±0.45^ |
|  | 15 | | 10^1.05±0.52^ | | - | | 10^0.23^ | | 10^1.08±0.58^ | | 10^0.99±0.67^ |
|  | 20 | | 10^1.66±0.13^ | | - | | 10^0.81±0.59^ | | 10^0.70^ | | - |
|  | 25 | | 10^1.07^ | | - | | - | | 10^1.08^ | | - |
|  | 30 | | - | | - | | - | | - | | - |
| Salivary glands of F1 field-collected *Aedes albopictus* | 0 | | - | | - | | - | | - | | - |
|  | 5 | | - | | - | | - | | 10^1.51±0.31^ | | 10^0.89±0.76^ |
|  | 10 | | - | | - | | 10^0.85±0.81^ | | 10^1.20±0.58^ | | 10^1.01±0.19^ |
|  | 15 | | - | | - | | 10^0.88^ | | 10^0.91^ | | - |
|  | 20 | | - | | - | | 10^0.49±0.47^ | | 10^0.14^ | | - |
|  | 25 | | - | | - | | - | | 10^0.18±0.19^ | | - |
|  | 30 | | - | | - | | - | | 10^0.12±0.05^ | | - |
| Head of F1 field-collected *Aedes albopictus* | 0 | | - | | - | | - | | - | | - |
|  | 5 | | - | | - | | 10^0.38±0.21^ | | 10^0.42±0.28^ | | 10^0.37±0.34^ |
|  | 10 | | - | | - | | 10^0.80±1.10^ | | 10^1.69±1.32^ | | 10^0.60±0.16^ |
|  | 15 | | - | | - | | - | | 10^0.22^ | | - |
|  | 20 | | - | | - | | - | | - | | - |
|  | 25 | | - | | - | | - | | - | | - |
|  | 30 | | - | | - | | - | | 10^0.80±0.98^ | | - |

**Additional file 1: Table S2** Detail virus titers of tested *Wolbachia* free F4 mosquitoes

| Samples tested |  |  |  | |  | |  | |  | |  |
| --- | --- | --- | --- | --- | --- | --- | --- | --- | --- | --- | --- |
|  | 0 dpi | 5 dpi | 10 dpi | 15 dpi | | 20 dpi | | 25 dpi | | 30 dpi | |
| Thorax-abdomen | 10^2.98±0.34^ | 0 | 10^2.29±0.37^ | 10^4.26±0.58^ | | 10^3.92±0.43^ | | 10^3.70±0.32^ | | 10^3.59±0.77^ | |
| Salivary glands | 0 | 0 | 10^1.02±0.12^ | 10^2.51±0.99^ | | 10^2.94±0.50^ | | 10^2.95±0.23^ | | 10^3.11±0.45^ | |
| Head | 0 | 0 | 10^1.52±0.26^ | 10^2.05±0.63^ | | 10^2.46±0.26^ | | 10^2.41±0.68^ | | 10^2.22±0.26^ | |


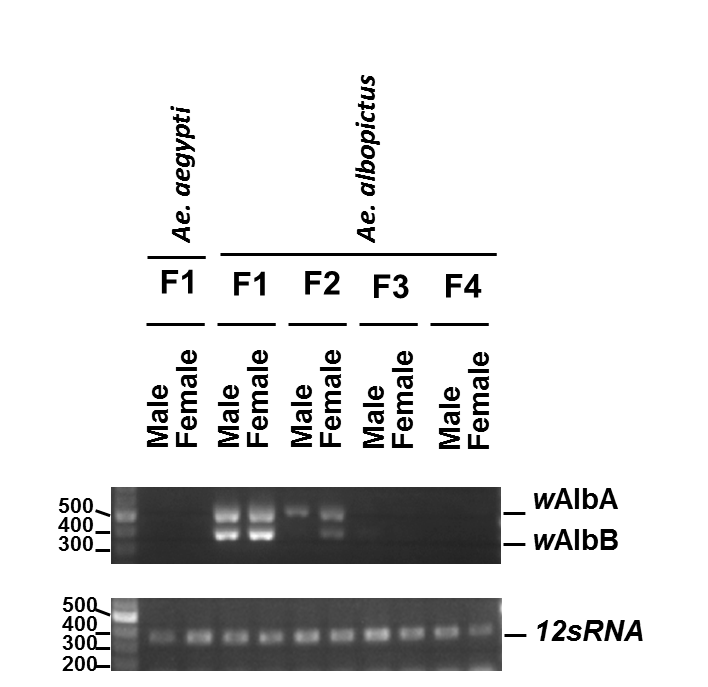


**Additional file 1: Figure S1** Generations of the *Wolbachia*-free *Ae. albopictus* mosquitoes. PCR was conducted to confirm the *Wolbachia* infection (*w*AblA and *w*AblB) with field captured mosquitoes. *Ae. albopictus* were treated with 1mg tetracycline for 3 generations. 12sRNA was used as internal control. Representative Agarose gel was shown.
